# Retrieval-Based Model Accounts for Striking Profile of Episodic Memory and Generalization

**DOI:** 10.1038/srep31330

**Published:** 2016-08-11

**Authors:** Andrea Banino, Raphael Koster, Demis Hassabis, Dharshan Kumaran

**Affiliations:** 1 Google DeepMind, 5 New St Square, London EC4A 3TW, UK; 2 Gatsby Computational Neuroscience Unit, 25 Howland St, London W1T 4JG, UK; 3 Institute of Cognitive Neuroscience, University College London, 17 Queen Square, WC1N 3AR, UK

## Abstract

A fundamental theoretical tension exists between the role of the hippocampus in generalizing across a set of related episodes, and in supporting memory for individual episodes. Whilst the former requires an appreciation of the commonalities across episodes, the latter emphasizes the representation of the specifics of individual experiences. We developed a novel version of the hippocampal-dependent paired associate inference (PAI) paradigm, which afforded us the unique opportunity to investigate the relationship between episodic memory and generalization in parallel. Across four experiments, we provide surprising evidence that the overlap between object pairs in the PAI paradigm results in a marked loss of episodic memory. Critically, however, we demonstrate that superior generalization ability was associated with stronger episodic memory. Through computational simulations we show that this striking profile of behavioral findings is best accounted for by a mechanism by which generalization occurs at the point of retrieval, through the recombination of related episodes on the fly. Taken together, our study offers new insights into the intricate relationship between episodic memory and generalization, and constrains theories of the mechanisms by which the hippocampus supports generalization.

The hippocampus is widely accepted to play a critical role in episodic memory, the capacity to remember individual experiences from the past (e.g. where one parked the car on a given day)^1^,^2^. However, recent evidence suggests that the hippocampus plays an important role across species in experimental paradigms where successful performance depends on exploiting the commonalities present across multiple related experiences^3^,^4^,^5^,^6^,^7^,^8^,^9^,^10^,^11^,^12^,^13^,^14^,^15^,^16^. Indeed, these findings provoke fundamental questions about the nature of hippocampal representations, and how putative mechanisms by which the hippocampus might support generalization in such scenarios fits with its well established role in episodic memory.

Two classes of mechanisms have been proposed to account for the role of the hippocampus in generalization: firstly, “encoding-based” models^11^,^17^,^18^ argue that the hippocampus integrates together related experiences (e.g. two object pairs A-B & B-C) at the point of encoding, resulting in the formation of blended representations (e.g. A-B-C: linking A, B and C) that directly support generalization at the time of test. In contrast, “retrieval-based” models^19^ (also see ref. 20) seek to retain a computational principle that is viewed to be critical to the functioning of an episodic memory system: pattern separation^21^,^22^,^23^,^24^,^25^,^26^,^27^,^28^,^29^, whereby even related experiences result in the formation of orthogonalized representations in the hippocampus during the study phase. Generalization, according to this view, represents an emergent phenomenon whereby multiple related episodic memory traces (e.g. coding for experiences A-B, and B-C) are re-activated and recombined within a memory space dynamically shaped at the point of retrieval (i.e. during test trials). The question of which of these two very different mechanisms provides a more accurate account of the hippocampal contribution to generalization remains an open question based on empirical evidence to date.

In this study, we employed a prototypical paradigm widely used to study the role of the hippocampus in generalization – the paired associate inference (PAI) task^3^,^4^,^5^,^30^,^31^ – to address these issues. Whilst the PAI paradigm has been used to test the mechanisms underlying generalization, the relationship between episodic memory for the individual associative experiences (i.e. A-B, B-C object pairs) and ability to generalize (i.e. appreciate the indirect relationship between A and C objects) has not been studied before. Here we develop an adapted version of the task that allowed us to obtain measures of participants’ episodic memory for the individual experiences as well as their ability to generalize (see Fig. 1). Importantly, the two classes of mechanisms discussed above suggest divergent predictions about the nature of the relationship between episodic memory and generalization: encoding-based models hold that the ability to generalize depends on blended representations (i.e. A-B-C) that imply a concomitant loss of episodic memory. In contrast, better memory for the individual episodes is associated with superior generalization performance in retrieval-based models, because robust reactivation of pattern separated episodic traces facilitates episodic recombination mediated by a recurrent mechanism. We conducted several studies to explore the effects of different experimental conditions – for example, whether participants knew during the study phase of the task that their ability to generalize would subsequently be tested – on the relationship between episodic memory and generalization.

## Results

For all experiments we report the results in a Bayesian statistical framework (see Methods for the explanations of the advantages of Bayesian statistic over null hypothesis significance test in the context of our experiments & e.g.^32^,^33^). Notably, the posterior distributions were summarised using the highest density interval (HDI), which is defined as the interval that covers a certain percentage of the distribution (in our case 95%) in a way that every point inside the interval has a higher credibility than any point outside it. The HDI is then used to obtain the set of credible values for a certain parameter, which can be used to make unbiased decisions on parameters’ values. For instance if the HDI of the estimated posterior distribution of a certain parameter include 0 then it is possible to infer that that parameter is not credibly different from 0. A similar concept in null hypothesis significance testing (NHST) is represented by confidence interval (CI), however this does not describe a probability distribution over parameters values, but merely defines two end points, which are based on the hidden intentions of the experimenter and not on prior knowledge (cf. stated prior distribution)^32^,^33^. We used standard methods for performing approximate bayesian inference (i.e. markov chain monte carlo (MCMC) algorithms: see Methods for details), in situations where calculating the exact posterior distribution is intractable. For all the models (across experiments) visual inspection of both trace and density plots revealed an almost perfect overlap between chains. The Gelman-Rubin test^34^ confirmed the convergence of the chains for each parameter (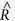
mean values were 1.00 for all parameters). Finally, the analysis of the autocorrelation function (ACF) and *effective sample size* (ESS)^35^ revealed that the MCMC were sufficiently large and accurate estimates of the posterior distributions. Note that in addition, for the purposes of clarity we also report the results of a classical statistical analysis for Experiment 1 (see Supplementary Information).

### Experiment 1

**Figure 1:**
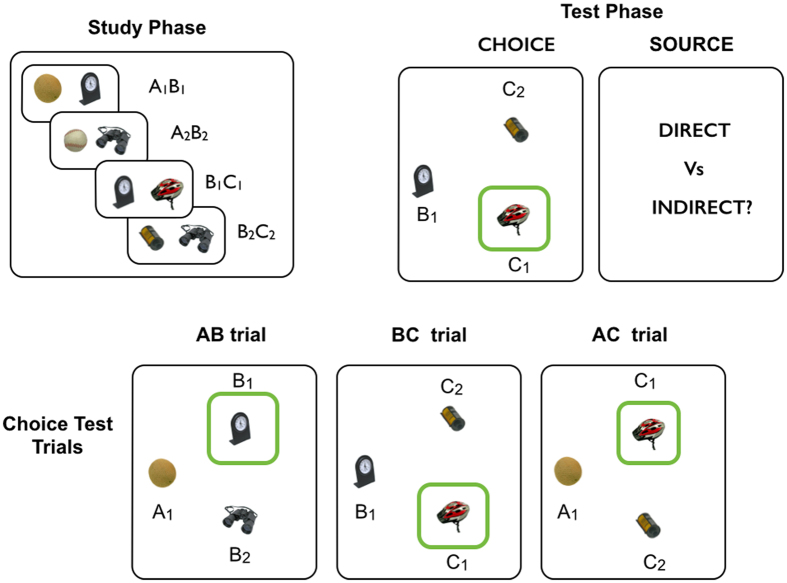
Experimental Design. During the study phase (top left panel) overlapping object pairs derived from triplets are presented (e.g. A1-B1, B1-C1). The test phase consists of two types of trials (top right panel): **choice test trials**: illustrated is a BC test trial, where participants should choose the target object (C1 (helmet) in this example), that is (indirectly) associated with the cue object (B1: clock). **Source test trials**: following each choice test trial, participants are asked whether the association between the cue (e.g. B1) and chosen object (e.g. C1) is “direct” (meaning that the two objects were actually presented as a pair during the study phase) or “indirect” (i.e. that the two objects were not presented together, but are indirectly associated via an unseen item). The correct response in a BC source trial would be “direct”, and in an AC trial “indirect”. Critically, source trials targeted participant’s episodic memory, for example testing whether they knew that B1 and C1 objects had actually been presented together. In contrast, participants’ responses during choice test trials probed their memory for whether objects were associated with one another (i.e. part of the same triplet), *regardless* of whether they had been presented together (e.g. B1-C1) or not (e.g. A1-C1). Bottom Panel: the three types of choice test trials (i.e. AB, BC, AC) are illustrated, each of which was followed by a source judgement (i.e. as depicted in top right panel). Object images were obtained from the Bank of Standardized Stimuli (BOSS) and are licensed under the Attribution-ShareAlike 3.0 Unported license (http://creativecommons.org/licenses/by-sa/3.0/).

**Figure 2:**
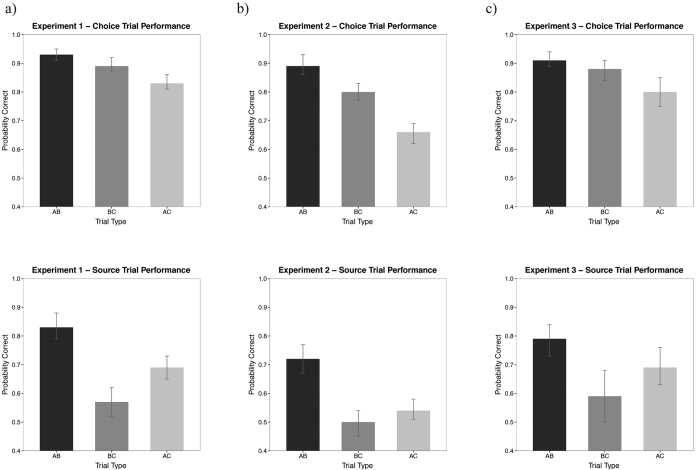
Performance on experiment 1 (**a**), experiment 2 (**b**) and experiment 3 (**c**) for choice trials and source trials (upper and lower panels respectively). Error bars indicate the 95% highest density interval (HDI) that contains the most credible 95% of the values.

During choice test trials (see Methods), participants were required to select which of two objects was associated with the probe object (e.g. whether B1 or B2 was associated with A1, in an AB choice test trial: see Fig. 1 and Methods). Their performance was highly proficient on AB trials [95% HDI from 0.91 to 0.95, mean of HDI = 0.93], and BC trials [95% HDI from 0.87 to 0.91, mean of HDI = 0.89] (see Fig. 2). Notably, they were also successful on choice test trials where an inference was required: AC trials [95% HDI from 0.81 to 0.86, mean of HDI = 0.83]. Interestingly, participants’ performance on AB choice trials was credibly better than on BC choice trials (i.e. zero was not among the credible values of the difference between the relevant posterior distributions [AB − BC: 95% HDI from 0.01 to 0.07, mean of HDI = 0.04].

Critically, our experimental design included source test trials that followed choice test trials which specifically probed the episodic nature of participant’s underlying representations, by asking them to judge whether the two relevant items (e.g. A1 and B1 on an AB trial) were “directly” associated (i.e. had been presented together during the study phase) or “indirectly” associated (i.e. were related through an intervening item: e.g. A1 and C1 on an AC trial: see Methods). In contrast, participants’ responses during choice test trials assessed their memory for whether objects were associated with one another (i.e. part of the same triplet), *regardless* of whether they had been presented together as part of a single episode (e.g. A1-B1) or not (e.g. A1-C1) (see Supplemental Information for further details on the distinction between choice and source trials).

Our results revealed a striking asymmetry between performance on AB and BC trials. Whilst participants performed successfully on AB source trials [95% HDI from 0.79 to 0.88, mean of HDI = 0.83], their performance on BC source trials was not far above chance level [95% HDI from 0.52 to 0.62, mean of HDI = 0.57]. Performance on AC source trials had a mean of the HDI equal to 0.69 [95% HDI from 0.65 to 0.73].

Notably, both performance on AB source trials (see above) and AC source trials was credibly different from performance on BC trials: for both comparisons the value of zero was not among the credible values of the HDI [AB − BC: 95% HDI from 0.18 to 0.32, mean of HDI = 0.26; BC − AC: 95% HDI from −0.18 to −0.05, mean of HDI = −0.11].

**Figure 3:**
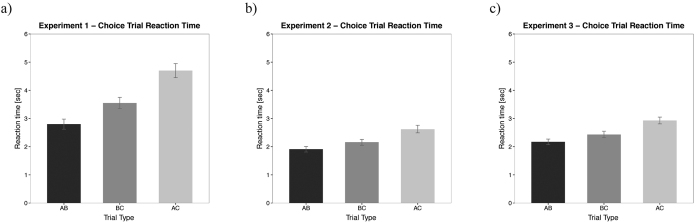
Reaction times for experiment 1 (**a**), experiment 2 (**b**) and experiment 3 (**c**) for choice trials. Error bars indicate the 95% highest density interval (HDI) that contains the most credible 95% of the values.

This profile of results was also reflected in our analysis of the reaction time (RT) data. Note that the data are reported on the original scale after the log-posterior distribution had been transformed. Participants were faster to respond on AB choice trials [95% HDI from 2.62 seconds to 2.98 seconds, mean of HDI = 2.80 seconds] than on both the BC choice trials [95% HDI from 3.35 seconds to 3.75 seconds, mean of HDI = 3.55 seconds] and AC choice trials [95% HDI from 4.45 seconds to 4.95 seconds, mean of HDI = 4.70 seconds] (see Fig. 3). The direct comparison between these conditions showed that participants’ RT in AB choice trials were credibly different from both BC and AC choice trials [AB − BC: 95% HDI from −1.02 seconds to −0.48 seconds, mean of HDI = −0.75 seconds; AB − AC: 95% HDI from −2.21 seconds to −1.60 seconds, mean of HDI = −1.90 seconds]. RTs on BC choice trials were credibly faster than on AC trials [BC − AC: 95% HDI from −1.48 seconds to −0.84 seconds, mean of HDI = −1.15 seconds].

We also analyzed the RT data relating to source judgments: this revealed a similar profile. Specifically, participants were faster on AB source trials than on BC source trials [AB − BC: 95% HDI from −0.20 seconds to −0.09 seconds, mean of HDI = −0.15 seconds]. RTs during AB source trials were also credibly lower than during AC source trials [AB − AC: 95% HDI from −0.13 seconds to −0.03 seconds, mean of HDI = −0.08 seconds]. Participants were also slower to respond on BC source trials than on AC trials [BC − AC: 95% HDI from 0.01 seconds to −0.12 seconds, mean of HDI = 0.07 seconds].

#### Logistic regression

The results described above provide evidence for a striking asymmetry between participants’ episodic memory – indexed by the performance on source test trials – whereby memory for A-B experiences is credibly stronger than memory for B-C experiences, the latter being close to chance levels. To explore the relationship between episodic memory (i.e. indexed source test trial performance) and generalization/inference (i.e. A-C choice test trial performance), we conducted a logistic regression analysis that included these critical variables (see Methods).

**Figure 4:**
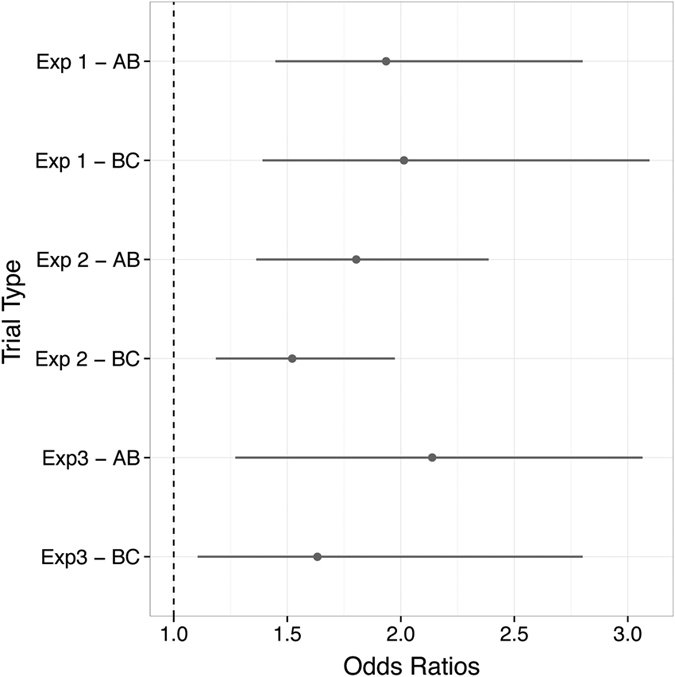
Odds ratios for AB source and BC source predictors for experiment 1, experiment 2 and experiment 3. The odds ratios represent the odds that participants will make a correct inference judgment (i.e. correct answer on AC choice trial) given correct performance on an AB (or BC) source trial (cf. incorrect performance on respective source trial).

The regressors coding for performance on AB source trials and BC source trials had a significant positive correlation with performance on AC choice trials [*β*_*ABsource*_: 95% HDI from 0.37 to 1.03, mean of HDI = 0.66 ; *β*_*BCsource*_: 95% HDI from 0.33 to 1.13, mean of HDI = 0.70] with all the other predictors held constant (see Fig. 4 for posterior odds ratios). Indeed, the magnitude of AB and BC predictors was similar suggesting that they contribute equally to the probability of making a correct inference on A-C choice test trials. Indeed, the comparison of their posterior distributions showed no significant differences between the magnitude of their coefficients [*β*_*ABsource*_ − *β*_*BCsource*_: 95% HDI from −0.47 to 0.47, mean of HDI = 0.02]. Also the predictor coding for BC choice trial showed a positive correlation with AC choice performance [*β*_*BCchoice*_: 95% HDI from 0.21 to 1.23, mean of HDI = 0.72], but this effect was not credibly different from the predictor coding for BC source [*β*_*BCchoice*_ − *β*_*BCsource*_: 95% HDI from −0.42 to 0.47, mean of HDI = 0.02]. As such, our findings imply that better episodic memory for both A-B and B-C trials is associated with superior A-C performance – a result that points towards retrieval-based models of inference (cf encoding-based – see discussion).

### Experiment 2

In Experiment 1, participants were instructed prior to the experiment that they would need to complete generalization trials (i.e. AC) and the experimental schedule was organized into study-test cycles. In Experiment 2, we asked whether a similar profile of results would be observed under different experimental conditions where participants were not aware during the study phase that their ability to generalize would be subsequently tested. Specifically, in this experiment participants completed all encoding sessions before test, and were in fact instructed to keep the object pairs (e.g. A1-B1, B1-C1) experienced during the study phase as separate as possible to avoid interference (see Methods). Despite these considerable differences in the set-up of the experimental paradigm, we observed a similar profile of results (see Fig. 2).

Participants’ performance on AB, BC and AC choice test trials was: [AB trials 95% HDI from 0.86 to 0.93, mean of HDI = 0.89; BC trials 95% HDI from 0.77 to 0.83, mean of HDI = 0.80; AC trials 95% HDI from 0.62 to 0.69, mean of HDI = 0.66]. The posterior mean comparison tests again revealed that participants’ performance on AB choice trials were credibly better than both BC choice trials [95% HDI from 0.04 to 0.14, mean of HDI = 0.09] and AC choice trials [95% HDI from 0.10 to 0.19, mean of HDI = 0.15] (see Fig. 2).

Performance on source test trials also showed a clear asymmetry between AB and BC test trials: [AB trials 95% HDI from 0.67 to 0.77, mean of HDI = 0.72; BC trials 95% HDI from 0.45 to 0.54, mean of HDI = 0.50]. Performance on AC source trials was slightly above chance [95% HDI from 0.51 to 0.58, mean of HDI = 0.54]. Notably, a mean comparison analysis revealed that performance on AB source trials were credibly different from both BC and AC source trials: in fact for both comparisons zero was not among the credible values of the HDI [AB − BC: 95% HDI from 0.16 to 0.30, mean of HDI = 0.23; AB − AC: 95% HDI from 0.12 to 0.24, mean of HDI = −0.18].

RT analysis during choice trials revealed that participants were faster on AB choice trials [95% HDI from 1.81 seconds to 2.01 seconds, mean of HDI = 1.91 seconds] than on both BC choice trials [95% HDI from 2.05 seconds to 2.26 seconds, mean of HDI = 2.16 seconds] and AC choice trials [95% HDI from 2.49 seconds to 2.76 seconds, mean of HDI = 2.62 seconds] (see Fig. 3). Participants were credibly faster in responding to AB choice trials (cf. BC and AC trials): [AB − BC: 95% HDI from −0.39 seconds to −0.10 seconds, mean of HDI = −0.25 seconds; AB − AC: 95% HDI from −0.87 seconds to −0.55 seconds, mean of HDI = −0.71 seconds]. Additionally, RT on BC choice trials was credibly faster than AC trials [BC − AC: 95% HDI from −0.63 seconds to −0.29 seconds, mean of HDI = −0.46 seconds].

Lastly, the analysis of source RT data revealed that participants were credibly faster on AB source trials than both BC and AC trials [AB − BC: 95% HDI from −0.09 seconds to 0 seconds, mean of HDI = −0.05 seconds; AB − AC: 95% HDI from −0.13 seconds to −0.03 seconds, mean of HDI = −0.08 seconds]. No difference was found between BC source trials than AC trials [BC − AC: 95% HDI from −0.09 seconds to 0.02 seconds, mean of HDI = −0.04 seconds].

#### Logistic regression

As in experiment 1, in a logistic regression analysis where AC test trial performance was the dependent variable, the predictors coding for performance on AB source trials and BC source trials were credibly different from zero: [*β*_*ABsource*_: 95% HDI from 0.31 to 0.87, mean of HDI = 0.59; *β*_*BCsource*_: 95% HDI from 0.17 to 0.68, mean of HDI = 0.42] with all the other predictors held constant (see Fig. 4 for posterior odds ratios). It is worth noting that although the coefficient for AB source performance was numerically higher than the coefficient for BC source performance, a comparison of their posterior distributions revealed that they were not credibly different [*β*_*ABsource*_ − *β*_*BCsource*_: 95% HDI from −0.24 to 0.58, mean of HDI = 0.16]. Furthermore, the predictor coding for BC choice showed positive correlation with AC choice [*β*_*BCchoice*_: 95% HDI from 0.36 to 0.55, mean of HDI = 0.46], a further comparison revealed that this predictor was not credibly from the predictor coding for BC source [*β*_*BCchoice*_ − *β*_*BCsource*_: 95% HDI from −0.33 to 0.47, mean of HDI = 0.04]. These findings provide further support that better episodic memory – even for BC trials where source performance was found to be at chance level across participants – is associated with superior generalization on AC choice test trials.

### Experiment 3

This experiment followed the structure of experiment 1 (i.e. encoding-test cycles), with the exception that a novel/familiar scene was presented before each BC trial (see Methods). The rationale for this manipulation was that there are theoretical proposals^36^ and empirical evidence^37^ that novelty/familiarity biases the hippocampal system towards an encoding/retrieval mode, respectively. A hypothesis consequent on this perspective in the PAI paradigm is that BC trials which are preceded by a novel scene may result in relatively more pattern separated representations for the relevant AB and BC experiences, and therefore a preservation of AB and BC episodic memory (i.e. indexed by source memory trials). In comparison, BC trials preceded by a familiar scene may trigger pattern completion (i.e. of the initial AB experience), integration/blending, and therefore relatively worse episodic memory for AB and BC experiences.

However, in our experiment we did not find credible effects of the novelty/familiarity manipulation – specifically, the familiarity factor (*β*_*2[k]*_) and its interaction with the trial category factor (*β*_*1*x*2[j,k]*_) were not credibly different from zero in any of the relevant analyses. As such, the results we report focus on the difference between trial types, effectively collapsing across the novelty/familiarity factor (see Methods), and therefore serve largely to replicate the findings of experiment 1.

Participants’ performance on choice test trials was: AB trials [95% HDI from 0.89 to 0.94, mean of HDI = 0.91], BC trials [95% HDI from 0.84 to 0.91, mean of HDI = 0.88], and AC trials [95% HDI from 0.75 to 0.85, mean of HDI = 0.80]. In this experiment participants’ choice performance on AB was numerically higher than that on BC trials, but not credibly different [AB − BC: 95% HDI from 0 to 0.08, mean of HDI = 0.04] (see Fig. 2).

Source data analysis confirmed the marked asymmetry between performance on AB and BC trials: [AB: 95% HDI from 0.73 to 0.84, mean of HDI = 0.79; BC trials 95% HDI from 0.50 to 0.68, mean of HDI = 0.59]. In contrast to BC source performance which did not differ from chance levels, performance on AC source trials was well above chance [95% HDI from 0.63 to 0.76, mean of HDI = 0.69]. Performance on AB source trials was credibly different from performance on BC source trials [AB − BC: 95% HDI from 0.18 to 0.32, mean of HDI = 0.26] (see Fig. 2).

Analysis of reaction times shown that participants were faster on AB choice trials [95% HDI from 2.07 seconds to 2.27 seconds, mean of HDI = 2.17 seconds] than on both BC choice trials [95% HDI from 2.32 seconds to 2.55 seconds, mean of HDI = 2.43 seconds] and AC choice trials [95% HDI from 2.81 seconds to 3.05 seconds, mean of HDI = 2.93 seconds]. Further, participants were credibly faster on AB choice trials than both BC and AC choice trials [AB − BC: 95% HDI from −0.47 seconds to −0.05 seconds, mean of HDI = −0.26 seconds; AB − AC: 95% HDI from −0.98 seconds to −0.52 seconds, mean of HDI = −0.75 seconds] and that the RT on BC choice trials was credibly lower than on AC trials [BC − AC: 95% HDI from −0.72 seconds to −0.24 seconds, mean of HDI = −0.49 seconds] (see Fig. 3).

Analysis of RT source data showed a similar pattern: participants were credibly faster on AB source trials than both BC and AC trials [AB − BC: 95% HDI from −0.21 seconds to −0.07 seconds, mean of HDI = −0.14 seconds; AB − AC: 95% HDI from −0.39 seconds to −0.25 seconds, mean of HDI = −0.32 seconds]. Also, participants were faster on BC source trials than on AC trials [BC − AC: 95% HDI from −0.24 seconds to −0.12 seconds, mean of HDI = −0.18 seconds].

#### Logistic regression

The categorical predictor coding for familiarity (*β*_*2[k]*_) was not credibly different from zero and hence we considered only the predictors coding for the different trial types. Both predictors for AB and BC source performance were positively correlated with AC test trial performance, as in the previous two experiments: [*β*_*ABsource*_: 95% HDI from 0.44 to 1.12, mean of HDI = 0.76; *β*_*BCsource*_: 95% HDI from 0.10 to 1.03, mean of HDI = 0.49] with all the other predictors held fixed (see Fig. 4 for posterior odds ratios). No significant difference was found between their magnitude: [*β*_*ABsource*_ − *β*_*BCsource*_: 95% HDI from −0.27 to 0.88, mean of HDI = 0.30]. Finally the predictor coding for BC choice trial shown a positive correlation with AC trial performance [*β*_*BCchoice*_: 95% HDI from 0.14 to 1.18, mean of HDI = 0.67], but this was not credibly different from the BC source predictor [*β*_*BCchoice*_ − *β*_*BCsource*_: 95% HDI from −0.30 to 0.57, mean of HDI = 0.19], as in the previous experiments.

#### Recognition memory

Participants’ showed above chance recognition memory for novel scenes [Mean corrected hit rate = 29%, SD = 16%]. Also, we tested participants’ performance on the main experimental test trials as a function of their subsequent recognition memory for test trials preceded by novel scenes. This analysis revealed no difference between choice test trials in which participants subsequently recognized the novel scene and trials in which they did not [AB_hit_ − AB_miss_: 95% HDI from −2.54 to 2.68, mean of HDI = 0.07, BC_hit_ − BC_miss_: 95% HDI from −2.55 to 2.60, mean of HDI = 0.06; AC_hit_ − AC_miss_: 95% HDI from −2.53 to 2.64, mean of HDI = 0.07; where “hit” and “miss” denote whether the scene was recognized or not]. The same pattern of result held for source test trials [AB_hit_ − AB_miss_: 95% HDI from −2.07 to 2.42, mean of HDI = 0.17, BC_hit_ − BC_miss_: 95% HDI from −2.00 to 2.47, mean of HDI = 0.17; AC_hit_ − AC_miss_: 95% HDI from −2.06 to 2.41, mean of HDI = 0.16].

### Experiment 4

Our findings across three experiments show a consistent profile of findings, marked by a clear asymmetry between performance on AB and BC source test trials. In this follow-up experiment (see Supplemental Information and Fig. S1 for details) – in which AC test trials were omitted for half of the triplets (i.e. there were only AB and BC test trials) – we considered and excluded the possibility that this effect might be driven by learning occurring during AC test trials which by design preceded AB and BC test trials (see Methods).

## Computational Modelling

**Figure 5:**
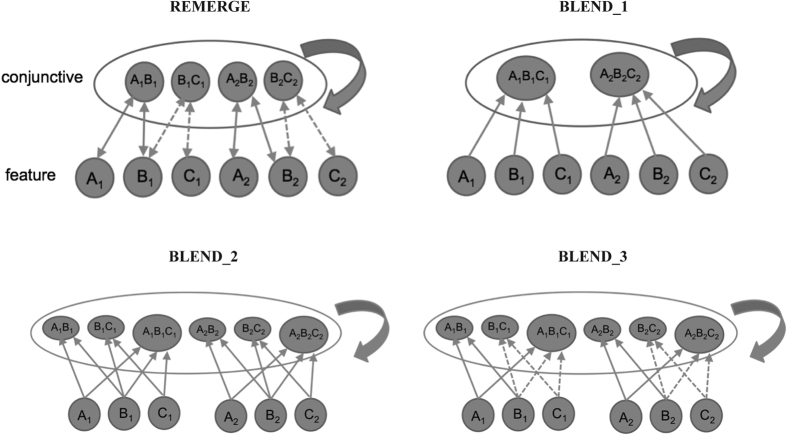
Schematic of computational models used to simulate performance in the PAI task: 2 triplets illustrated (A_1_B_1_C_1_, A_2_B_2_C_2_). REMERGE: recurrent excitatory connections between feature and conjunctive layers. Curved arrow indicates competitive inhibition (i.e. softmax function) in conjunctive layer in all models. Dashed lines indicate weaker weights (cf. solid lines), implementing the assumption of weaker encoding of BC, as compared to AB, object pairs. Blend_1: the simplest model incorporating the notion of the formation of blended/integrated representations during encoding (i.e. coded by a single ABC unit). All Blend models have feedforward feature-to-conjunctive excitatory connections only. Blend_2: in addition to Blend_1, an extra AB and BC unit are included. Blend_3: in addition to Blend_2, a weight asymmetry is present denoted by dashed lines (i.e. as in REMERGE) to denote weaker encoding of BC pairs. See Methods section for details.

We next sought to provide a mechanistic account of the striking profile of behavioural findings observed across the three experiments. In particular, we pitted a retrieval-based model of generalization^19^ directly against encoding-based mechanisms^11^,^17^,^18^ that were constructed within the same connectionist framework (see Fig. 4). Intuitively, these models represent a highly simplified abstraction of the processing within the hippocampal system (e.g.^19^): broadly, the feature layer, where units denote individual objects (e.g. object A1), can be related to the entorhinal cortex and the conjunctive layer to the CA3 region of the hippocampus, where units are conjunctive (e.g. A1B1 unit). The conjunctive units implement an idealization of the notion of pattern separated representations for overlapping episodes (e.g. A1B1, B1C1 – see Fig. 5). The retrieval-based REMERGE model implements a principle of big-loop recurrence within the hippocampal system, whereby the output of the system can be fed back in as a new input, through bidirectional excitatory connections between the feature and conjunctive layer^19^. This allows generalization, even between distantly related experiences, to occur through memory space that is dynamically constructed at the point of retrieval. In contrast, encoding-based models^11^,^17^,^18^ do not have recurrent connections, but implement the notion that blended/integrated representations than span episodes are formed during encoding through units on the conjunctive layer (e.g. A1B1C1). We examine variants of both classes of models that additionally encompass the assumption of proactive interference during BC encoding.

### Model Architectures

#### REMERGE model

As shown in Fig. 4, units in the conjunctive layer (e.g. A_1_B_1_, B_1_C_1_) correspond to object pairs (e.g. A_1_-B_1_, B_1_-C_1_) presented during the studied pairs. Two triplet pairs (i.e. A_1_-B_1_-C_1_, A_2_-B_2_C_2_) are shown^15^,^19^. The feature layer is connected to the conjunctive layer by bidirectional excitatory connections, and individual units denote individual objects (e.g. A_1_, B_1_). External input is presented to the feature layer (e.g. A_1_, C_1_, C_3_ on an AC test trial). Activity of feature units was determined by a logistic function. Processing in the network continued through an iterative constraint-style satisfaction process, mediated by the recurrent connections. The curved arrow indicates inhibitory competition between conjunctive units, implemented by the standard softmax function. The conjunctive layer connects to the response layer (not shown in figure) through feedforward weights that are the same as the feature-conjunctive weights, such that activation of the A_1_B_1_ conjunctive unit drives choice of the B_1_ object. A softmax function operating on response unit activities determined the network’s choice in a given trial. For a more detailed description of the model see ref. 19, and a schematic description of its operating principles is given in ref. 15. To capture the asymmetry between AB and BC test trial performance, we made the assumption that proactive interference occurred during BC encoding resulting in a slight decrement in weight strength (see below).

The 3 free parameters in this model used to simulate *choice test trial* performance were: i) the magnitude of weights between AB conjunctive units (e.g. A_1_B_1_) and A and B feature layer units (e.g. A_1_, B_1_). ii) The reduction in weight strength between BC conjunctive units and B and C feature units. iii) The network temperature used across feature, conjunctive and response layers.

Following previous work^27^, we simulated performance on *source test trials* through a measure that captured the amount of mismatching activity present on the feature layer (***x*** in equation (1)) – where the presence of mismatching recall provides evidence that an input pattern has not been actually experienced before. Intuitively, an A_1_C_1_ trial would induce significant activation through recurrence of the B_1_ feature unit – leading the network to make an “indirect” response. As such, two additional free parameters, common to all 4 models, were included: iv) a threshold value against the amount of mismatching activity was compared (***thresh***) v) ***τ***_***source***_ - a temperature specific to source judgments (see equation (1)).


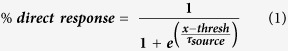


#### BLEND models

These models were implemented as described for the REMERGE model (e.g. logistic function on feature layer etc), except where stated. Importantly, these were feedforward models (i.e. feature→ conjunctive, with no recurrence) designed to implement the principle of integrated/blended representations of encoding-based models through conjunctive units (e.g. A_1_B_1_C_1_) which were connected to all 3 feature units for a given triplet (i.e. A_1_, B_1_, C_1_). Note that although recurrence was not present in these blend networks, one backward pass (from conjunctive→ feature layer) was allowed to compute the level of mismatching activity on the feature layer (i.e. as in ref. 27). In order of increasing complexity (see Fig. 5):

Blend_1 model has 4 free parameters (denoted as (iii, iv, v) in REMERGE above) plus a free parameter specifying magnitude of feedforward weights from all 3 feature units (i.e. A_1_, B_1_, C_1_) to the “blended” conjunctive unit (e.g. A_1_B_1_C_1_).

Blend_2 model incorporates additional AB and BC units in the conjunctive layer (i.e. as well as the integrated ABC unit), as if there are pattern separated representations for individual episodes as well as a blended representation. It has the 4 free parameters of blend_1 plus one additional parameter (i.e. parameter (i) of REMERGE above).

Blend_3 model incorporates the 5 free parameters of Blend_2, plus an additional parameter to allow for the assumption of proactive interference during BC encoding (i.e. parameter (ii) of REMERGE above: see Fig. 5).

### Model Results

Given the highly similar profiles of the choice and source data in experiments 1–3, we focus here on simulating the observed data in experiment 1. We performed a hyperparameter sweep for all networks (i.e. REMERGE and the 3 versions of the blend model), to identify the parameters that best fit the empirical data in terms of the choice and source data across the group of subjects. We report the results of the relevant indices (averaged across subjects): i.e the negative log likelihood (NLL)) and BIC^38^ in Table 1 (see below).

**Figure 6:**
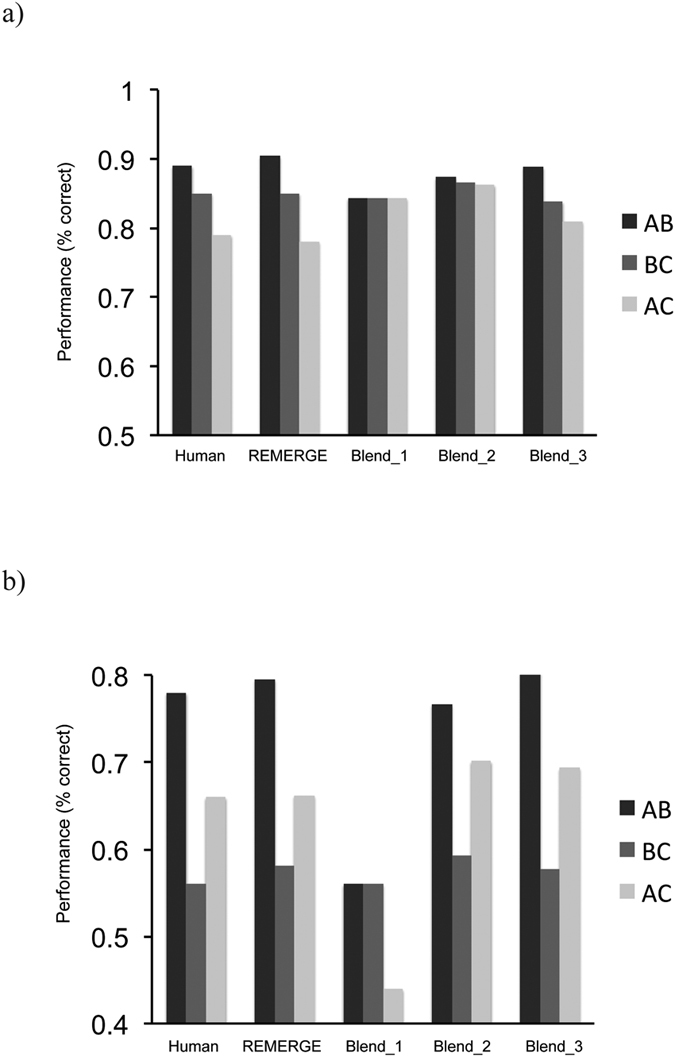
Illustration of fit of models to empirical data for choice (**a**) and source test trials (**b**) from Experiment 1. The REMERGE model provides a good qualitative fit to the profile of empirical data, and the best quantitative fit (see Table 1). See Fig. 5 and section on model architectures for details of model specification.

As shown in Fig. 6, REMERGE – as described in ref. 19, but incorporating the assumption of a degree of proactive interference during BC encoding through a slight weight asymmetry (see Methods) – was able to reproduce the key features of the empirical data: i.e. the slightly superior of AB (cf BC) choice performance, and the striking asymmetry between AB and BC source judgments, with the latter being near chance levels. REMERGE also produced the best quantitative fit (see Table 1), with the BIC index providing evidence in favour of it over the next best fitting model (i.e. blend_3)^39^. Notably, the simplest interpretation of the encoding based hypothesis – the blend_1 model – where a single integrated representation (i.e. represented by a single ABC unit) is formed during encoding of the overlapping A-B and B-C pairs, provided a poor qualitative and quantitative fit to both choice and source data (see Fig. 6 and Table 1). The only blend model that produced a reasonable qualitative and quantitative fit to the data (i.e. blend_3) can be considered a specific instantiation of an encoding-based of model – since it incorporated both the notion of proactive interference during encoding, and maintained individual representations for AB and BC episodes (see Discussion).

## Discussion

Despite rising evidence that the hippocampus plays an important role in certain types of generalization^13^,^15^, it remains unclear whether this is primarily a result of representations formed at the stage of encoding^11^,^17^,^18^ or mechanisms operating at the point of retrieval (i.e. at test)^14^. Here we took the PAI paradigm^3^,^4^,^5^,^30^,^31^,^40^, a widely used hippocampal-dependent task, and modified it to enable us to study the relationship between episodic memory and generalization. Through combining computational modeling with this richer behavioral dataset, we aimed to reveal the underlying mechanisms that support generalization.

Results across 4 separate studies involving different experimental conditions demonstrated that participants’ ability to generalize (i.e. perform inferences on AC choice test trials) was associated with a surprising loss of episodic memory. Whilst participants’ performance was superior on AB as compared to BC choice test trials, the asymmetry in terms of episodic memory performance was much more pronounced: performance on BC source judgments was near chance levels, with a relative preservation of AB episodic memory. Interestingly, however, despite the poor episodic memory overall on BC trials logistic regression analyses across all experiments (Fig. 4) revealed that better BC (and indeed AB) episodic memory was associated with a superior capacity for generalization.

Computational modeling demonstrated the ability of the retrieval-based model REMERGE to provide the best qualitative and quantitative fit to the empirical data among the models tested (see Fig. 6 and Table 1). Indeed, the finding from the logistic regression that superior episodic memory for BC (and AB) pairs relates to better generalization performance is also consistent with a retrieval-based model of generalization – because robust reactivation of pattern separated episodic traces facilitates episodic recombination mediated by a recurrent mechanism (see ref. 19 for simulation of this effect). Notably, the only alteration to the original model^19^ was the assumption of a degree of proactive interference during BC encoding resulting in weaker encoding of BC, as compared to AB, object pairs. This notion of proactive interference – which relates to a wider literature on the associative learning of overlapping pairs^41^,^42^,^43^ was implemented in the model as slightly stronger weights between the A and B feature units and AB conjunctive units, as compared to B and C feature unit to BC conjunctive unit weights (see Methods).

In contrast, the simplest model incorporating the notion of the formation of blended/integrated representations during encoding^11^,^13^,^17^ (i.e. coded by a single ABC unit in the blend_1 model), provided a poor fit to the qualitative profile of the data (see Fig. 5). The most complex version of the blend model (i.e blend_3) was able to provide a reasonable qualitative fit to the data, though the quantitative fit was substantially worse than REMERGE (see Table 1) – despite being able to incorporate the assumption of stronger encoding of AB pairs than BC pairs through an additional AB unit as well as a similar weight asymmetry to that used in REMERGE (see Methods and Fig. 5). It is also worth noting that this model can be viewed as a specific instantiation of an encoding-based model that preserves pattern separated representations for individual episodes, whilst allowing for the creation of a new blended representation. As such, this model configuration would appear to differ from current proposals of encoding-based models (e.g.^11^,^17^,^18^,^40^). Moreover, this representational scheme is consistent with proposed extensions of the REMERGE model that allow for the creation of “stored generalizations” (see ref. 19) that would coexist with representations of individual episodes.

The results of these simulations – and additionally the profile of reaction times (i.e. AB < BC < AC) – favours a retrieval-based (cf. encoding-based) mechanism such as REMERGE^19^, in which overlapping episodes (i.e. AB, BC object pairs in the PAI paradigm) are represented in a pattern separated fashion, with related episodes being recombined at the point of retrieval to support generalization. However, a natural question is what explains the pronounced loss of BC episodic memory in the context of such putatively pattern separated representations. To illustrate this, it is worth considering what occurs during source trials in the REMERGE model: during a BC source test trial there is partial activation of the A object unit on the feature layer, due to the slightly weaker encoding of the BC object pair based on the assumption of proactive interference. It is this partial activation of feature units denoting objects not actually present on the screen (i.e. A unit in BC source trial), that translates into a mismatching recall signal (cf.^27^). This mismatching recall signal leads the network to assign a relatively high probability (i.e. around 50%) that the objects (i.e. B & C) weren’t actually experienced as a pair in one episode. This mismatching recall signal arises to a much lesser extent in AB source trials due to the stronger encoding of AB pairs which through competitive inhibition in the conjunctive layer effectively curtails activation of the BC unit (and therefore activation of the C unit on the feature layer). Note that it is for similar reasons that high levels of mismatching recall (i.e. partial activation of the B unit) during an AC source trial causes the network to assign a lower probability of objects A and C having been studied together (i.e. ~30% in experiment 1) – and therefore relatively high levels of accuracy (i.e. ~70% correct in experiment 1). To summarize: the simulations demonstrate that the loss of BC episodic memory does not imply the existence of blended representations. Instead, the observed episodic memory loss can be accounted for by mismatching recall, in the context of pattern separated representations for the individual pairs.

Our simulations were set up to directly compare the fit of the retrieval-based REMERGE model to the behavioral data to a range of increasingly complex encoding-based models with blended/integrated representations, all implemented within the same architectural framework. Whilst the results of the quantitative model fitting procedure (i.e. BIC values: see ref. 44) provide strong evidence that the empirical data is best captured by the retrieval-based REMERGE mechanism, it is important to note that we have not tested the full space of models that incorporate the notion of blended representations (e.g. the temporal context model (TCM)^18^,^45^). Indeed, previous research on the PAI paradigm suggests that encoding and retrieval-based mechanisms may both operate (e.g.^13^). In particular a recent multivariate fMRI study^31^ (also see ref. 46) provided evidence that in the anterior hippocampus the representation of individual items (i.e. A and C objects) becomes more similar following exposure to overlapping pairs (i.e. AB, BC) during the study phase of the PAI paradigm – whereas item representations within the posterior hippocampus became less similar. Whilst it was not possible to link these representational differences to generalization at the behavioral level – because participants were intentionally trained to ceiling levels of performance – one hypothesis would be that both encoding-based and retrieval-based mechanisms support generalization, with a differential contribution of anterior and posterior hippocampus, respectively.

Encoding and retrieval based mechanisms may also play differential roles depending on the experimental conditions (e.g.^13^). It is interesting to note, however, that at least in our study we observed the same qualitative profile of findings across 4 experiments across a range of experimental conditions. In experiments 1 and 4, which involved encoding-test cycles, participants encoded object pairs having been instructed that they would be subsequently tested on their ability to generalize. In experiment 2, however, participants were told to keep the object pairs as separate as possible so as to perform as well as possible on a subsequent episodic memory test (i.e. the generalization test was a surprise). In experiment 3, we manipulated the novelty/familiarity of scenes that preceded BC encoding trials with the aim of biasing hippocampal processing towards encoding/retrieval mode, respectively^36^ – drawing inspiration from a previous study that showed significant effects on generalization^37^. Whilst we did not find an effect of novelty/familiarity – either as a main effect or as a function of subsequent memory – one should be cautious in interpreting this null finding, particularly given the significant differences between paradigms.

The PAI paradigm has been widely used to study the mechanisms by which the hippocampus supports generalization. Our study, however, represents the first to relate episodic memory in the PAI task to the ability to generalize. Indeed, existing paradigms have tended to study episodic memory for overlapping associations (e.g.^41^,^42^,^43^), or generalization^4^,^30^,^31^,^40^ in isolation from one another. Our study, therefore, reveals new aspects of the widely used PAI paradigm and shows that an apparent loss of episodic memory occurs alongside an ability to generalize. Further, our findings demonstrate that the combination of pattern separated episodic representations and recurrence implemented in the REMERGE model can account for the complex relationship we discovered between episodic memory and generalization.

## Methods

### Participants

Healthy individuals who were free from neurological or psychiatric disease, and currently undertaking or had recently completed a university degree at the University College London took part in the experiments (experiment 1: n = 24 (15 females); experiment 2: n = 21 (13 females); experiment 3: n = 16 (11 females) experiment 4: n = 16 (6 females). All participants gave full informed consent prior to the experiment. All experimental procedures were approved by the local research ethics committee (Division of Psychology and Language Sciences, University College London), and procedures were carried out in accordance with the approved guidelines. Participants were paid a minimum of £10 for completing the experiment, and an additional amount (i.e. up to £10) depending on their performance on the task.

### Object stimuli

Object stimuli were a selection of pictures obtained from the Bank of Standardized Stimuli (BOSS - https://sites.google.com/site/bosstimuli), which is a database of photo stimuli that have been normalized across several parameters^47^. All objects were presented in the RGB colour space (8 Bits/pixel) on a white background, with a resolution of 600 × 600 dpi and they were all resized to a width and a height of 220 pixels.

### Scene stimuli

A set of 80 landscape pictures was obtained from various sources on the internet to serve as novel/familiar stimuli in Experiment 3. All the pictures depicted a landscape scenario, without any prominent objects. All scenes were presented in the RGB colour space (8 Bits/pixel), with a resolution of 72 × 72 dpi and they were all resized to a width and a height of 500 pixels using Photoshop^©^ CS5.

### Procedures common to all 3 experiments

In all three experiments, 80 different object triplets (e.g. A1-B1-C1, A2-B2-C2) were used. The allocation of stimuli into triads and the sessions order were randomized across participants.

### Encoding Trials

During study trials participants viewed a pair of objects (e.g. A1-B1, B1-C1) each displayed on either side of the screen. The left-right position of the objects on the screen was pseudo-randomised across trials and each pair was presented for 2.5 seconds. A fixation cross, presented for 0.5 second, preceded the presentation of the next trial (Fig. 1).

### Test Trials

#### Choice trials

Participants were presented with three objects; a *cue* was presented on the top-centre of the screen (e.g. object A1) and two possible choices were presented on either side at the bottom of the screen (e.g. C1 and C7). To control for familiarity, the incorrect choice was a familiar item (i.e. C7), which was a member of a different triplet. As in previous studies (e.g.^40^), there were 2 types of choice test trials: those that required generalization (i.e. associative inference: A-C choice test trials), and those that did not (i.e. A-B and B-C choice test trials). For example, in an A-B choice test trial: object A1 could be presented at the top of the screen, with the subject required to choose B1 (correct) over B2 (incorrect) object stimuli. Participants had a maximum of 10 seconds to respond, by pressing the left or the right arrow key buttons, corresponding to the item at the bottom (e.g. B1) that they thought was associated with the cue (e.g. A1). After participant’s choice, a blue bar appeared below the chosen item (duration 1 second), regardless of its correctness (Fig. 1B).

#### Source trials

This type of trial was a novel feature of our PAI paradigm, and not incorporated in previous studies (e.g.^40^). Critically, source trials provided a measure of participant’s episodic memory, for example testing whether they knew that A1 and B1 objects had actually been presented together. In contrast, participants’ responses during choice test trials probed their memory for whether objects were associated with one another (i.e. part of the same triplet), *regardless* of whether they had been presented together (e.g. A1-B1) or not (e.g. A1-C1).

Source trials were implemented as follows (see Fig. 1): after a participant’s response during a given choice test trial, participants were asked to state whether the chosen object had actually been presented with the cue object (i.e. was “directly” associated), or whether it was “indirectly” associated through a third object. As such, on an A-C test trial, an “indirect” response would be appropriate, whilst in A-B and B-C test trials “direct” responses would be correct. This phase was also self-paced, with a maximum of 10 seconds given for participants to make the direct/indirect judgement. A fixation cross, presented for 0.5 second, preceded the presentation of the next trial.

### Specifics of each experiment

Prior to the start of each experiment, participants completed a short demo (involving different object stimuli) that was specific to each experiment: for example in experiments 1 & 3, this informed them that there would be encoding-test cycles. In experiment 2, participants were shown a demo relating to how to perform test trials after the last encoding session.

#### Experiment 1

Each participant was presented with 80 different triplets divided into 5 encoding-test cycles containing 16 triplets each (i.e. mirroring the design of ref. 40). The allocation of stimuli into triplets and the sessions order were randomized across participants. Each encoding session was composed of 32 trials: 16 AB trials followed by 16 BC trials, in pseudorandom order. Each object pair (e.g. A1-B1) was presented once. At the end of each encoding session, participants completed 16 AB and 16 BC choice and source test trials, as well as 16 AC choice and source test trials. The order of test trials was pseudorandom, except for the constraint (i.e. as in previous studies e.g.^40^) that inference test trials (e.g. involving objects A1 and C1) preceded test trials involving the corresponding AB and BC objects (i.e. A1-B1 and B1-C1).

#### Experiment 2

As in Experiment 1, participants were presented with 80 different triplets divided into 5 sessions of 16 triplets each. The critical difference between experiment 1 was that test trials occurred at the end of the experiment (i.e. instead of encoding-test cycles). Further, participants were instructed to remember each association (e.g. A1-B1 and B1-C1) *in isolation* specifically to avoid interference between related pairs (i.e. they were told that their memory would be tested for each object pair at the end of the session, though testing in fact included inference trials as in experiment 1).

Each encoding session was composed of 32 associative trials, 16 AB trials followed by 16 BC trials and each association (e.g. A1-B1) was repeated twice. Furthermore we ensured a minimum of 10 trials gap between the presentation of one AB trial and its related BC association. At the end of the last encoding session, participants completed 80 AB and 80 BC choice and source test trials, as well as 80 AC choice and source test trials, in pseudorandom order (except for the constraint that inference test trials preceded A-B and B-C test trials). The schedule of test trial presentation was also designed to keep the gap between study and test trials approximately equated across triplets. Choice and source test trials were self-paced as in experiment 1, but the maximum time given to respond was 5 seconds (rather than 10 seconds).

#### Experiment 3

Prior to the main experiments, participants completed a one-back task where they viewed a series of 40 scene stimuli presented in the centre of the screen for 3 seconds, followed by a fixation cross (duration 0.5 seconds). Each scene was presented twice. These 40 scenes were subsequently used as “familiar” stimuli in the main experiment. Participants were asked to press the spacebar if the same picture repeated, which occurred on a small % of trials. Each block of forty pictures was repeated 5 times. The allocation of landscape pictures into the novel, the familiar or the foils set was randomized across participants.

The schedule of study and test trial presentation (i.e. in terms of 5 encoding test-cycles, 1 repetition of each AB and BC object pair etc.) was exactly as for experiment 1, with the exception that a scene stimulus (2 second duration) was presented before each BC trial which was followed by a fixation cross (1.5 second duration). Half of BC trials were preceded by a familiar scene stimulus (i.e. a stimulus presented during the one-back task) and half were preceded by novel scenes. Participants were told to pay attention to the scene stimuli, but not that there would be a subsequent memory test.

Scene memory recognition test: at the end of the main experimental session participants were tested on their memory for the 40 novel scene stimuli (20 novel scene foils were included). They were asked to determine whether each picture was old or new, and rate their confidence in their decision (on a 6 point scale: 1 – new sure, 2 – new unsure, 3 – new guess, 4 – old guess, 5 – old unsure, 6 – old sure). Recognition memory trials were self paced (10 seconds max duration), with order pseudo-randomised across participants.

#### Experiment 4

This experiment was conducted in the same fashion as experiment 1, but with one exception: for half of the triplets, AC choice and source test trials were *omitted* (i.e. there were only AB and BC source test trials for these triplets). The order of AB and BC test trials was pseudorandomised and balanced across triplets, such that ½ AB trials were preceded by BC trials (and vice versa).

## Bayesian Analysis

All the statistical analyses were conducted using Bayesian data analysis that, for the research designs implemented throughout these studies, present four main advantages over NHST. Firstly, Hierarchical Bayesian analysis (HBA) naturally accommodates the structure of the sampled data and does not require making any additional assumptions as for traditional NHST ANOVA (e.g. normally distributed data within groups and equal variances across the groups)^32^,^48^,^49^,^50^. A second advantage of Bayesian methods is that HBA guarantees unbiased estimate of parameters that is not dependent on sampling and testing procedures, but only on the prior distributions chosen by the experimenter, which are usually based on previous results and are clearly stated^32^,^33^. Furthermore, Bayesian approaches result in the estimation of the full posterior probability distribution of the parameters, which are more informative than single point estimates obtained in traditional NHST^50^. Moreover, the posterior distributions can be summarized using the highest density interval (HDI) such that values inside the 95% of the HDI are more credible than outside values (i.e. they have higher probability density). HDI can be used to make unbiased decisions on parameters’ values: for instance when performing a mean comparison test, if zero is within the HDI then the two compared values cannot be considered as being credibly different. A similar concept in NHST is represented by the confidence interval (CI): however this only defines two end points, which are based on the intentions of the experimenter and not on prior knowledge^32^,^33^. Finally, in a Bayesian setting multiple comparisons do not require any correction; in fact, each comparison can be simply seen as a different viewpoint on the posterior distribution^48^.

## Model specifications

All the models were run using the R statistical computing software (http://www.R-project.org)^51^ and JAGS^52^, a Markov chain Monte Carlo (MCMC) Gibbs sampling algorithm. Diagnosis on MCMC samples were performed using the Coda package^53^.

### Trials exclusion criteria

For choice judgments all trials were considered in the analyses; whereas, for source judgments all trials for which the relative choice judgments were not correct were excluded from the analyses.

### Hierarchical Bayesian models for parameter estimations

Individual trial *i* are denoted as *y*_*i|j,s*_to indicate cases within subject *s* and category *j*. Trials in each category are considered as multiple independent dichotomous events drawn randomly from a binomial distribution with *p*_*i|j,s*_ being the probability of success of subject *s* in category *j* and *N*_*i|j,s*_ representing the total number of trials performed by the subject in each category (equation (2)). Probabilities *p*_*i|j,s*_are estimated to be distributed as a beta distribution with mode *ω*_*j,s*_ and a concentration parameter *k*_*j*_ (equation (3)). The mode *ω*_*j,s*_follow a logistic function with a baseline, *β*_*0*_, plus a deflection for the category*, β*_*j*_, and a main effect for subjects, *β*_*s*_, to account for the within-subject design of the experiments. The concentration parameter *k*_*j*_ is given a vague gamma prior. The baseline, *β*_*0*_, is given a normal prior distribution with mean zero and a fixed standard deviation. For experiments 1 and 2, the category and subject deflections parameters, *β*_*j*_and *β*_*s*_, are given a normal prior with a mean of zero to account for the fact that each deflection parameter is supposed to sum to zero. The scales of the deflections, σ_βj_ and σ_βs,_ are estimated from data and are described using a t-folded distribution with fixed hyper parameters^48^,^54^,^55^ (equation (4)). For experiment 3 a second nominal predictor, *β*_*k*_, was added to account for the difference between novel and familiar trials and the interaction between *β*_*j*_and *β*_*k*_was also considered (equation (5)). The prior distribution on *β*_*k*_ was again normal with a mean of zero and the scale parameter estimated from a folded-t distribution with fixed hyper parameters.


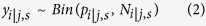



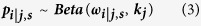



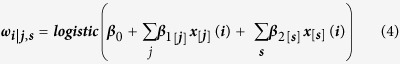



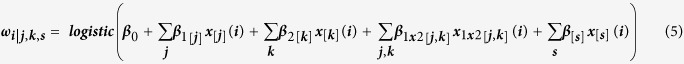


#### MCMC details

For each analysis we run five chains with a thinning of 5 steps. A total of 60,000 samples per chain were drawn after 1000 adaptive steps and 2000 burn-in samples. For each parameter under analysis MCMC chains were diagnosed for both representativeness and accuracy of posterior distributions. Specifically, to test the former we relied on visual examination of the MCMCs trajectories by analysing chains overlapping in both trace and density plots, but we also relied on the Gelman-Rubin statistics^34^. To test the accuracy of the estimation of the posterior we evaluated the autocorrelation function (ACF), but also on a statistic called the *effective sample size* (ESS)^48^,^50^.

### Hierarchical Bayesian models for reaction times

Observation have been log-transformed, and the trial category is again a nominal predictor with 3 levels, AB (direct paired trials), BC (direct paired trials) and AC (inference trials). The model was fitted with a predictor to account for the subjects’ main effect (see above for details). Individual trial are denoted as *y*_*i|j,s*_to indicate cases within subject *s* and category *j* (equation (6)). Trials in each category are assumed to be drawn randomly from a t-distribution with central tendency *μ*_*i|j,s*_ (equation (7)). The scale parameter of the t-distribution, *σ*_*j*,_ is estimated for each single category *j* and it is described by a t-folded distribution with fixed hyper parameters. The normality parameter, *v*, of the t-distribution is given a broad exponential prior. The baseline, *β*_*0*_, is describe by a normal prior distribution with mean of zero and a standard deviation, σ_0_, made broad depending on the standard deviation of the observations^48^. The category and subject deflections parameters, *β*_*j*_and *β*_*s*_, are given a normal prior with a mean of zero to account for the fact that each deflection parameter is supposed to sum to zero. The scales of the deflections, σ_βj_ and σ_βs,_ are estimated from data and are described using a t-folded distribution with fixed hyper parameters^50^. For experiment 3 a second nominal predictor, *β*_*k*_, was added to account for the difference between novel and familiar trials and the interaction between *β*_*j*_and *β*_*k*_was also considered (equation (8)).


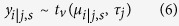



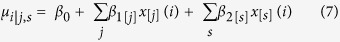



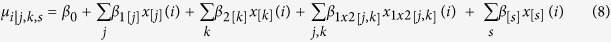


### MCMC details

For each analysis we run five chains with a thinning of 5 steps. A total of 85,000 samples per chain were drawn after 1000 adaptive steps and 2000 burn-in samples. For details about MCMC diagnostics see above.

### Hierarchical Bayesian logistic regression model

We also conducted a regression analysis to estimate subjects’ probability of correctly answering choice inference trials (AC_choice_) as a function of their correctness on BC choice trials and source judgments on AB_source_ (direct paired), BC_source_ (direct paired) and AC_source_ (indirectly paired) trials. The predictor for AB choice trials was excluded from the model due to colinearity with the AB source predictor. Individual trials are denoted as *y*_*i|s*_to indicate the i^th^ observation within subjects *s* (equation (9)). Trials are considered as multiple independent dichotomous events and so they are assumed to be drawn randomly from a binomial distribution with *p*_*i|s*_ being the probability of success of subject *s* on trial *i* and *N*_*i|s*_ equals 1 to denote that *y*_*i*_ = 1 with probability *p*_*i|s*_ and zero otherwise. The probability of subjects being correct is equal to the inverse logit of the category predictors plus a main effect for subjects (equation (10)). For experiment 3, an additional categorical predictor, *β*_k(i)_, was added to account for the difference between novel and familiar trials (equation (11)). All the predictors were given a normal prior with a mean of zero and a broad standard deviation. By assuming that the standard deviation of the normal distribution ranges from −100 to +100 the model specifies very little prior information about the value of the coefficients^50^. The model was also run with different priors (e.g. folded-t) and it showed almost null sensitivity to these changes.


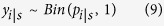



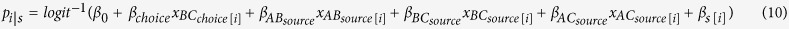



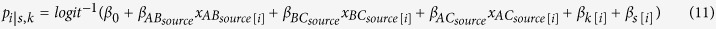


### MCMC details

For each analysis we run five chains with a thinning of 5 steps. A total of 85.000 samples per chain were drawn after 1000 adaptive steps and 2000 burn-in samples. For details about MCMC diagnostic see above.

## Additional Information

**How to cite this article**: Banino, A. *et al*. Retrieval-Based Model Accounts for Striking Profile of Episodic Memory and Generalization. *Sci. Rep.* **6**, 31330; doi: 10.1038/srep31330 (2016).

## Supplementary Material

Supplementary Information

## Figures and Tables

**Table 1:** Summary of model fits.

Model	NLL	Num params	BIC
Remerge	250	5	547
Blend_1	268	4	582
Blend_2	254	5	560
Blend_3	252	6	555

*Note NLL (negative log likelihood) of base (i.e. random) model is 332 (BIC = 664). Best fit parameters obtained by hyperparameter sweep (see Methods). BIC values are per participant. The BIC difference between REMERGE and the next best model (Blend_3) is 8, providing strong evidence for REMERGE (see ref. 44). See Fig. 5 and section on model architectures for details of model specification.
