# Supplementary material for: Retrieval-Based Model Accounts for Striking Profile of Episodic Memory and Generalization
Source: Sci Rep. 2016 Aug 11;6:31330. doi: 10.1038/srep31330 (PMC4980665; doi:10.1038/srep31330)

Retrieval-Based Model Accounts for Striking Profile of Episodic Memory and Generalization

Andrea Banino[1], Raphael Koster[1], Demis Hassabis[1,2], Dharshan Kumaran* [1,3].

[1]*Google DeepMind, 5 New Street Square, London EC4A 3TW, UK.*

[2]*Gatsby Computational Neuroscience Unit, 25 Howland St, London W1T 4JG, UK.*

[3]*Institute of Cognitive Neuroscience, University College London, 17 Queen Square, WC1N 3AR, UK.*

*Correspondence: Dharshan Kumaran dkumaran@google.com

Supplementary Information.

Difference between Choice and Source test trials in probing underlying episodic representations

Our motivation in this study was to develop a sensitive test of the extent to which participants possess episodic representations of the individual study episodes (e.g. A1-B1, B1-C1). Notably, participants' responses during choice test trials assessed their memory for whether objects were associated with one another (i.e. part of the same triplet), *regardless* of whether they had been presented together as part of a single episode (e.g. A1-B1) or not (e.g. A1-C1). Whilst successful performance on an AB (or BC) choice test trial must derive from previous episodic experience (e.g. a A1-B1 episode), this does not imply that the *underlying representation* used by the participant is episodic in nature. Concretely, if a participant had formed a single composite/blended *non-episodic* representation (e.g. A1-B1-C1) during the encoding phase, they could perform successfully on AB, BC and AC choice test trials. It is worth noting that this scenario arises in the paired associate inference task due to the inclusion of AC choice test trials where participants are required to choose the relevant C item (e.g. C1). In contrast, in a typical associative recognition memory experiment, AC type trials would be a lure trial requiring a "reject" (or "new") judgement – in such a setting, successful performance (i.e. "old") on AB and BC trials would indeed imply underlying episodic representations.

Performance on choice test trials, therefore, does not specifically probe the episodic nature of the representations. In contrast, successful performance across the set of source test trials – for example rendering an AB test trial to be "direct" and an AC test trial to be "indirect" – does require underlying episodic representations. Hence, we

emphasize performance on source trials as a purer window into the underlying episodic nature of the representations.

Experiment 1: Results of classical statistical analysis.

A one-way repeated measure ANOVA revealed a significant main effect of trial type, $F(2,28) = 33.18 – p = 1.2e-09$. The analysis confirmed that participants showed high performance on AB choice trials ($M = 0.89$, $SD = 0.11$) and BC choice trials ($M = 0.84$, $SD = 0.11$) and they were also proficient on trial were an inference was required (AC choice trial $M = 0.79$ $SD = 0.13$). The subsequent mean comparison analysis, adjusted for multiple comparisons using Bonferroni correction, confirmed that participants' performance on AB choice trial was significantly better than on BC choice trials (BC–AB = – 0.04, $p = 6.49e-04$).

Regarding the analysis of participants' episodic memory a one-way within subject ANOVA revealed a significant main effect of trial type, $F(2,46) = 41.85 – p = 4.42e-11$. Also, the asymmetry revealed by the Bayesian analysis was confirmed here. Participants' performed successfully on AB source trials ($M = 0.75$, $SD = 0.18$), but their performance on BC source trials was not significantly different than chance level ($M = 0.56$, $SD = 0.18$, $t(23) = 1.683$, p(2-tailed) = .106).

Of note, we conducted null hypothesis significance testing for all the experiments: results were in accordance with those of the Bayesian analysis, so the latter are only reported for the rest of the experiments.

Results of Experiment 4.

This experiment was conducted to exclude the possibility that the superior performance on AB source trials (cf BC source trials) was driven by learning during AC test trials: i.e. one could respond "direct" on a subsequent AB source trial simply by remembering that one has seen item (e.g.) A1 on a previous AC trial. Whilst one could argue that an analogous strategy could support successful BC source trial performance (i.e. respond "direct" if one has seen item (e.g.) C1 before on a previous AC test trial), this follow-up experiment specifically tests the "learning-at-test-time" hypothesis.

This experiment was conducted in the same fashion as experiment 1, but with one exception: for half of the triplets, AC choice and source test trials were *omitted* (i.e. there were only AB and BC source test trials for these triplets). This enabled us to directly compare performance in AB and BC source test trials, under conditions when the possibility of learning within AC test trials had been excluded.

To anticipate the key findings: we found the same marked asymmetry between AB and BC source test trial performance *with AC test trials excluded* that paralleled our finding in the previous experiments (i.e. mean of HDI for AB, BC source performance was 78% and 53% respectively). Indeed, BC source performance was not credibly different from chance level regardless of whether AC test trials were included/excluded. These results, therefore, demonstrate that the asymmetry between AB and BC source performance cannot be explained by learning during AC test trials.

For the condition in which AC trials were included in the test phase participants' performance on AB was higher than on BC choice test trials: [AB trials 95% HDI from 0.81 to 0.89, mean of HDI = 0.85; BC trials 95% HDI from 0.74 to 0.82, mean of HDI = 0.78], also they shown good performance on inference trials [AC trials 95% HDI from 0.69 to 0.81, mean of HDI = 0.75]. For the condition where AC trials were excluded from the test phase the performance on AB choice trial was higher than on BC trial [AB trials 95% HDI from 0.83 to 0.89, mean of HDI = 0.87; BC trials 95% HDI from 0.76 to 0.84, mean of HDI = 0.79]. For both condition the posterior mean comparison tests revealed no credible difference between AB and BC performance. [AB – BC (AC included): 95% HDI from 0 to 0.10, mean of HDI = 0.04; AB – BC (AC excluded): 95% HDI from 0 to 0.10, mean of HDI = 0.06] (see Figure 2). Also, the predictor coding for the two different conditions (i.e. AC trial included vs. AC trial excluded) was not credibly different from zero [95% HDI from -0.17 to 0.09, mean of HDI = -0.04] and the interaction coefficient was not credibly different from zero [95% HDI from -0.39 to 0.12, mean of HDI = -0.12].

Critically, performance on source test trials showed a clear asymmetry between AB and BC test trials in both conditions. Specifically, when AC source trials were excluded, participants shown higher performance on AB then BC source trials [AB trials 95% HDI from 0.73 to 0.84, mean of HDI = 0.78; BC trials 95% HDI from 0.48 to 0.62, mean of HDI = 0.53] and the mean comparison analysis revealed that this difference was credible [AB – BC: 95% HDI from 0.17 to 0.28, mean of HDI = 0.23]. Also they shown good performance on AC source trials [AB trials 95% HDI from 0.64 to 0.77, mean of HDI = 0.70] and this was credibly different from BC source performance [BC – AC: 95% HDI from -0.31 to -0.11, mean of HDI = -0.21]. When AC source trials

were included, participants proved better performance for AB then BC source trials [AB trials 95% HDI from 0.71 to 0.81, mean of HDI = 0.75; BC trials 95% HDI from 0.47 to 0.58, mean of HDI = 0.52] and the following mean comparison proved this difference to be credible [AB – BC: 95% HDI from 0.11 to 0.28, mean of HDI = 0.20]. Notably, it is worth highlighting that for both conditions BC source trials were not credibly different than chance level – i.e. their HDI include both 0.50. Also, the predictor coding for the two different conditions (AC trial included vs. AC trial not included) was not credibly different from zero [95% HDI from -0.20 to 0.10, mean of HDI = -0.02] as well as the interaction term [95% HDI from -0.55 to 0.04, mean of HDI = -0.25].

RT analysis during choice trials, when AC trials were excluded, revealed that participants were faster on AB choice trials [95% HDI from 2.38 seconds to 2.71 seconds, mean of HDI = 2.52 seconds] than BC choice trials [95% HDI from 2.77 seconds to 3.20 seconds, mean of HDI = 2.97 seconds]. The mean comparison analysis revealed that this faster responding in AB choice trials (cf. BC) was significant: [AB – BC: 95% HDI from -0.63 seconds to -0.26 seconds, mean of HDI = -0.44 seconds]. For the condition in which AC were included in the test, participants were again faster to answer AB then BC trials [95% HDI from 2.36 seconds to 2.70 seconds, mean of HDI = 2.54 seconds; 95% HDI from 2.78 seconds to 3.17 seconds, mean of HDI = 2.99 seconds] and this difference was credible [AB – BC: 95% HDI from -0.62 seconds to -0.26 seconds, mean of HDI = -0.44 seconds]. Additionally, neither the predictor coding for the inclusion of inference trials [95% HDI from -0.07 seconds to 0.05 seconds, mean of HDI = 0.00] nor the interaction predictor [95% HDI from -0.03 seconds to 0.23 seconds, mean of HDI = 0.10] were significant.

Lastly, the analysis of source RT data revealed that participants were faster on AB source trials than both BC trials when AC trial were included [AB: 95% HDI from 0.44 seconds to 0.57 seconds, mean of HDI = 0.50 seconds; BC: 95% HDI from 0.56 seconds to 0.75 seconds, mean of HDI = 0.65 seconds], and this difference was credibly different, [AB-BC: 95% HDI from -0.21 seconds to -0.07 seconds, mean of HDI = -0.14 seconds]. Also for the condition in which AC test trial were excluded participants were faster on AB then BC trials [AB: 95% HDI from 0.49 seconds to 0.54 seconds, mean of HDI = 0.55 seconds; BC: 95% HDI from 0.62 seconds to 0.79 seconds, mean of HDI = 0.70 seconds] and this difference was credible [AB-BC: 95% HDI from -0.22 seconds to -0.07 seconds, mean of HDI = -0.15 seconds]. Moreover, the predictor coding for the inclusion of inference trial was not significant [95% HDI from -0.04 seconds to 0.19 seconds, mean of HDI = 0.07] as the interaction term [95% HDI from -0.15 seconds to 0.32 seconds, mean of HDI = 0.08].

List of figures.

Figure S1. Performance on experiment 4 for choice trials and source trials. Error bars indicate the 95% highest density interval (HDI) that contains the most credible 95% of the values.

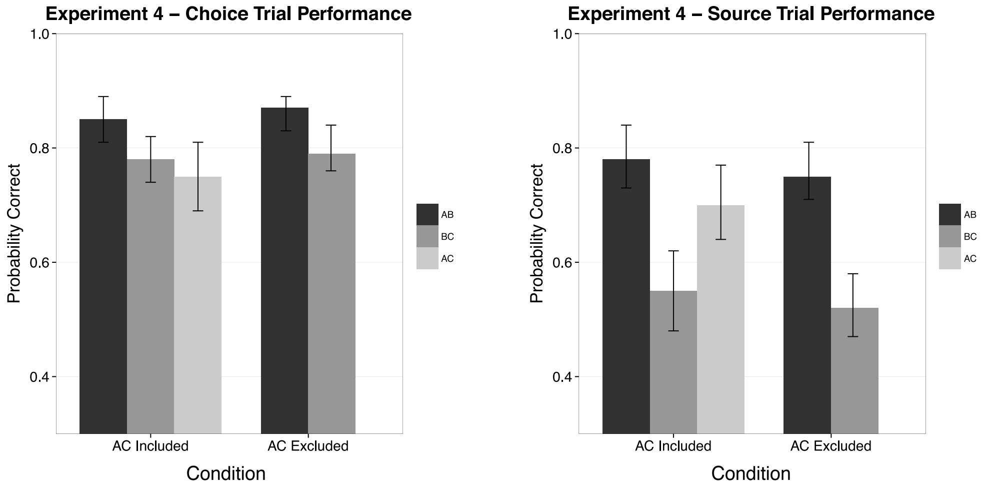

Supplement: Supplementary Information [file srep31330-s1.pdf]
